# Outcomes of Patients with Metastatic Colorectal Cancer Treated with Trifluridine/Tipiracil beyond the Second Line: A Multicenter Retrospective Study from Saudi Arabia

**DOI:** 10.1155/2022/3796783

**Published:** 2022-09-12

**Authors:** Mohammed Alghamdi, Shouki Bazarbashi, Mervat Mahrous, Omar Alshaer, Ahmed Mostafa Gad, Mohamed Aseafan, Mai Abdelgelil, Redhwan Mohammed Alshabi, Hosam Ali Alghanmi, Nasser Ahmed Naser, Husam Al Hariri, Abdulaziz ALHamad, Khalid Al-Saleh, Nashwa Abdel-Aziz, Sherif Elsamany

**Affiliations:** ^1^Department of Medical Oncology, King Saud University Medical City, King Saud University, Riyadh, Saudi Arabia; ^2^Department of Medical Oncology, Oncology Centre, King Faisal Specialist Hospital and Research Centre, Riyadh, Saudi Arabia; ^3^Oncology Department, Prince Sultan Military Medical City, Riyadh, Saudi Arabia; ^4^Department of Internal Medicine, Section of Medical Oncology, Security Forces Hospital, Riyadh, Saudi Arabia; ^5^Clinical Oncology and Nuclear Medicine Department, Faculty of Medicine, Ain Shams University, Cairo, Egypt; ^6^Clinical Oncology and Nuclear Medicine Department, Faculty of Medicine, Assiut University, Assiut, Egypt; ^7^Oncology Center, King Abdullah Medical City, Makkah, Saudi Arabia; ^8^Department of Medical Oncology, National Cancer Institute, Cairo University, Cairo, Egypt; ^9^Department of Medical Oncology, South Egypt Cancer Institute, Assiut University, Assiut, Egypt; ^10^Oncology Center, Mansoura University, Mansoura, Egypt

## Abstract

**Background:**

The outcome of patients with refractory metastatic colorectal cancer (mCRC) treated with trifluridine/tipiracil (FTD/TPI) beyond the second-line has not been studied in Saudi Arabia. Therefore, this multicenter retrospective analysis was conducted to evaluate the efficacy of FTD/TPI.

**Methods:**

This multicenter retrospective analysis included five centers in Saudi Arabia. FTD/TPI was administered to all the patients beyond the oxaliplatin- and irinotecan-based chemotherapy regimens. The electronic medical records were reviewed, and progression-free survival (PFS) and overall survival (OS) were determined.

**Results:**

The study included 100 patients with a mean age of 55.4 ± 11.8 years. The overall response to FTD/TPI was 4%. The median PFS was 4 months (95% confidence interval (CI) 3.487–4.513), and the median OS was 11 months (95% CI, 9.226–12.771). In a Cox regression analysis of the independent predictors for PFS, advanced stage of the disease (*P* = 0.037; HR, 2.614; and CI, 1.102–7.524), presence of lymph node metastasis (*P* = 0.018; HR, 3.664; and 95% CI, 1.187–8.650), and >2 metastatic sites (*P* = 0.020; HR, 1.723; and 95% CI, 1.089–2.727) were independent factors predicting disease progression. The Cox regression analysis confirmed that age ≥ 55 years (*P* = 0.046; HR, 1.667; and 95%, 1.097–3.100), advanced disease stage (*P* = 0.044; HR, 1.283; and 95% CI, 1.035–2.940), prior use of adjuvant chemotherapy (*P* = 0.037; HR, 0.892; and 95% CI, 0.481–0.994), liver metastasis (*P* = 0.025; HR, 2.015; and 95% CI, 1.091–3.720), >2 metastatic sites (*P* = 0.038; HR, 1.248; and 95% CI, 1.036–1.846), development of neutropenia after receiving first cycle of FTD/TPI (*P* = 0.042; HR, 1.505; and 95% CI, 1.064–2.167), and increased number of FTD/TPI cycles (*P* = 0.002; HR, 0.769; and 95% CI, 0.664–0.891) were independent variables for OS.

**Conclusion:**

Treatment with FTD/TPI is feasible and effective in daily clinical practice in Saudi Arabian patients. The risk of progression increased with advanced disease stage, lymph node metastasis, bone metastasis, and metastasis to >2 sites. Age ≥ 55 years, advanced disease stage, liver metastasis, metastasis to >2 sites, neutropenia after the first cycle of FTD/TPI, and increased number of FTD/TPI cycles were independent factors predicting mortality.

## 1. Background

Colorectal cancer is one of the most common causes of cancer-related mortalities [1]. In Saudi Arabia, many patients present at a young age, and the incidence has increased over the past 15 years [2]. In a significant number of patients, metastatic colorectal cancer (mCRC) is refractory, and the patients are able to receive more lines of therapy. FOLFOX, FOLFIRI, and FOLFOXIRI are well-established first-line therapies for mCRC [3]. Cetuximab and panitumumab are both FDA-approved agents for the first-line treatment of nonmutant mCRC. No inferiority or superiority was identified in the phase III ASPECCT study between the two drugs [4]. A multicenter phase II trial (the CHRONOS trial) of anti-EGFR rechallenge therapy with panitumumab guided by monitoring of the mutational status of RAS, BRAF, and EGFR in circulating tumor DNA (ctDNA). Results showed that disease control rate was obtained in 16/27 (59%, 95% CI: 41-78%) patients [5].

An interesting result from the KEYNOTE-177 trial suggests the efficacy of the first-line pembrolizumab monoclonal antibody that targets the programmed cell death protein 1 (PD-1) pathway in patients with microsatellite unstable disease. The results showed a trend toward reduced risk of death with pembrolizumab (hazard ratio (HR) 0.74 and 95% confidence interval (95% CI) 0.53–1.03) [6].

Patients with proficient DNA mismatch repair/microsatellite stable tumors who did not respond to both oxaliplatin and irinotecan/fluoropyrimidine combination, with or without anti-EGFR, have an unmet need for third-line effective therapy. Residual toxicity and lack of response to previously introduced chemotherapeutic agents and biologic drugs are considered limiting factors for drug rechallenge [1, 7]. Treatments in this setting include the use of regorafenib and trifluridine/tipiracil (FTD/TPI) [8, 9]. The introduction of the antihuman epidermal growth factor receptor 2 (HER2) antibody, trastuzumab, or trastuzumab plus the HER2 dimerization inhibitor, pertuzumab, for HER2-positive mCRC patients has shown promising effects [10].

The RECOURSE trial was a randomized, double-blind, placebo-controlled, international, phase III trial. Patients were randomly assigned, in a 2 : 1 ratio, to receive FTD/TPI or placebo and stratified according to RAS status and the time between first diagnosis of metastases and randomization. The study showed that FTD/TPI improved the survival of refractory mCRC patients who had failed chemotherapy with oxaliplatin, irinotecan, and molecular targeted agents (OS, 7.1 vs. 5.3 months; HR 0.68; and 95% CI, 0.58–0.81). [11]

There is a lack of data providing guidance for the best selection and sequencing of oxaliplatin- and irinotecan-based regimens for stage IV colorectal cancer. Different factors, including performance status, age, tumor sidedness, biomarkers (such as RAS and BRAF), and microsatellite instability, can be used to guide treatment selection [1, 12]. FTD and TPI were approved by the Saudi Food and Drug Authority in 2018 (supplementary file (available here)). To the best of our knowledge, the efficacy of FTD/TPI treatment beyond second-line treatment in patients with refractory stage IV CRC has not been studied in Saudi Arabia [13]. Therefore, we conducted this multicenter retrospective study to evaluate the efficacy of FTD/TPI beyond second-line treatment.

## 2. Materials and Methods

### 2.1. Study Population and Design

The current study was a multicenter retrospective analysis that included five centers in Saudi Arabia. Enrolment criteria included patients aged ≥18 years, biopsy-documented adenocarcinoma of the colon or rectum, stage IV colon or rectal cancer, and chemotherapy with oxaliplatin, irinotecan, and fluoropyrimidine. All patients received FTD/TPI beyond the oxaliplatin- and irinotecan-based chemotherapy regimens at a dose of 35 mg/m^2^ twice daily on days 1–5 and 8–12 every 28 days between January 2018 and August 2020. Institutional review board (IRB) approval was obtained from all participating centers. No informed consent was obtained as per the IRB roles for retrospective studies.

Electronic medical records were reviewed as appropriate. Data were collected anonymously, and confidentiality was maintained. Different variables were collected, including the demographic and clinical characteristics of the patients, pathologic features, stage, site of the disease, number of metastatic sites, number of metastatic lesions, type and line of palliative chemotherapy, type and line of biologic treatment, number of FTD/TPI cycles and duration of treatment, and incidence and grade of neutropenia after the first cycle of FTD/TPI. The response to FTD/TPI was evaluated using the revised response evaluation criteria in solid tumor (RECIST) guidelines [14]. Progression-free survival (PFS) and overall survival (OS) were calculated.

### 2.2. Statistical Analysis

Data were verified, coded by the researcher, and analyzed using SPSS version 24. Multivariate logistic regression analysis was performed to investigate the significant factors influencing the disease outcome (odds ratio (OR), 95% CI, and likelihood ratio test (LRT)). The Kaplan–Meier curve was used to estimate the median survival time. The log-rank test was used to compare the survival curves between the categories of explanatory variables. Multivariate Cox hazard regression analysis was performed to investigate the significant factors influencing OS and DFS (HR and 95% CI). Statistical significance was set at *P* < 0.05. PFS was defined as the time from starting treatment with FTD/TPI to disease progression or death due to any cause and/or the date of last follow-up. OS was defined as the interval from the date of starting treatment with FTD/TPI to death or the date of the last follow-up.

## 3. Results

### 3.1. Demographic Data and Tumor Characteristics

In total, 107 patients met enrollment criteria. Seven patients were excluded because of incomplete data. Analysis of 100 patients revealed a mean age of 55.4 ± 11.8 years (range, 29–85 years). Forty-two (42%) patients were female, and 58 (58%) were male. Overall, 8%, 53%, 36%, and 3% of the patients had an Eastern Cooperative Oncology Group performance status (ECOG PS) of 0, 1, 2, and 3, respectively. Wild-type *KRAS* was detected in 44% of the patients. N-ras, B-raf, and MSI data were not available for all patient cohorts. Sixty-four percent of the patients were diagnosed as having de novo metastasis, while the remaining patients progressed from the early stage. Thirty-six patients received chemotherapy in the adjuvant settings, seven patients received the FOLFOX regimen, 28 received XELOX, and only one received a single agent (capecitabine). Sigmoid was the most common site of cancer (36% of patients), followed by the rectum (27%) and right colon (21%). The liver was the most common site of metastasis (51%), followed by the lungs (41%), regional lymph nodes (32%), and peritoneum (18%). More than two metastatic sites were encountered in 46 (46%) patients. The number of the metastatic lesions was >5 in 63 (63%) patients (Table 1).

### 3.2. Previous Lines of Therapy

Oxaliplatin-based therapy was the most commonly used regimen (42%) for first-line chemotherapy, whereas irinotecan-based therapy was the most commonly used regimen for second-line chemotherapy (60%). For the first-line biological agents, bevacizumab was the most frequently used agent (33%), followed by cetuximab (25%). Bevacizumab was also the most frequently used second-line (49%) and third-line (16%) biological agent. Neutropenia of all grades was detected in 45% of patients after receiving the first cycle of FTD/TPI. Grade IV neutropenia was detected in 5% of patients. None of the patients experienced febrile neutropenia (Table 1).

### 3.3. Response to FTD/TPI

The median number of FTD/TPI cycles was 4 (range, 1–13). Of the total 100 patients, 41% received FTD/TPI as a third-line treatment, while 59% received it beyond the third-line treatment (fourth or fifth line).  Table 2 shows the response to FTD/TPI treatment. The overall response rate was 4%. One patient (1%) had a complete response (CR), while 3 (3%), 28 (28%), and 68 (68%) patients had partial response (PR), stable disease (SD), and progressive disease (PD), respectively (Table 2).

### 3.4. Survival Analysis

**Figure 1:**
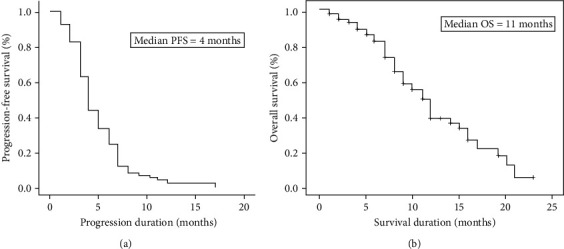
Kaplan–Meier survival curves. (a) Progression-free survival (PFS); the median PFS is 4 months (95% confidence interval (CI), 3.487–4.513). (b) Overall survival (OS); the median OS is 11 months (95% CI, 9.226–12.771).

During the study, 54 (54%) patients died, with a median follow-up of 15 months. The median PFS was 4 months (95% CI, 3.487–4.513), while the median OS was 11 months (95% CI, 9.226–12.771). Figures 1(a) and 1(b) show the Kaplan–Meier curves of PFS and OS of the studied patients, respectively.

In a Cox regression analysis of the independent predictors for PFS, the advanced stage of the disease (*P* = 0.037; HR, 2.614; and 95% CI, 1.102–7.524), lymph node metastasis (*P* = 0.018; HR, 3.664; and 95% CI, 1.187–8.650), bone metastasis (*P* = 0.036; HR, 2.790; and 95% CI; 1.073–8.221), and more than two sites of metastasis (*P* = 0.020; HR, 1.723; and 95% CI, 1.089–2.727) were found independent predictors of disease progression. By contrast, prior adjuvant chemotherapy (*P* = 0.037; HR, 0.892; and 95% CI, 0.481–0.994), the development of neutropenia after receiving first cycle of FTD/TPI (*P* = 0.041; HR, 0.738; and 95% CI, 0.425–0.924), and increasing number of FTD/TPI cycles (*P* = 0.006; HR, 0.899; and 95% CI, 0.833–0.969) were factors associated with low risk of progression (Table 3).

The evaluation of the mortality predictors using logistic regression analysis and Cox regression analysis confirmed a significant increase in the risk of death at age ≥ 55 years (*P* = 0.046; HR, 1.667; and 95%, 1.097–3.100), advanced disease stage at initial diagnosis (*P* = 0.044; HR, 1.283; and 95% CI, 1.035–2.940), liver metastasis (*P* = 0.025; HR, 2.015; and 95% CI, 1.091–3.720), number of metastatic sites > 2 (*P* = 0.038; HR, 1.248; and 95% CI, 1.036–1.846). In addition, prior use of adjuvant chemotherapy (*P* = 0.037; HR 0.892; and 95% CI, 0.481–0.994), the development of neutropenia after receiving first cycle of FTD/TPI (*P* = 0.039; HR, 0.633; and 95% CI, 0.384–0.894), and increasing the number of FTD/TPI cycles (*P* = 0.002; HR, 0.769; and 95% CI, 0.664–0.891) were independent predictors of better OS (Tables 4 and 5).

## 4. Discussion

To the best of our knowledge, this is the first study to evaluate disease outcomes in patients with refractory mCRC who received FTD/TPI beyond second-line treatment in Saudi Arabia. The efficacy and safety of FTD/TPI monotherapy in adult patients with refractory mCRC were demonstrated in the phase III RECOURSE trial [11], and a significant improvement in OS was reported for patients treated with FTD/TPI beyond second-line treatment. Improvement of OS from 5.3 months with placebo to 7.1 months with FTD/TPI was achieved, and HR for death in the FTD/TPI group versus the placebo group was 0.68 (95% CI, 0.58–0.81; *P* < 0.001). A second phase III trial with an entirely Asian population, the TERRA study, confirmed these results [15]. The results of the current study show that treatment with FTD/TPI beyond oxaliplatin- and irinotecan-based chemotherapy has some efficacy. The overall response rate was 4%, and overall disease control (CR, PR, and SD) was 32%. It is noteworthy that FTD/TPI was administered as a third-line treatment in 41% of patients and beyond the third-line treatment in 59% of patients. This overall response to FTD/TPI in a cohort treated outside the context of a preplanned trial appears to be higher than that reported by the RECOURSE study [11]. The small number of patients included in our analysis may have accounted for the magnified response results. Moreover, ethnic differences may have affected their responses. Our results show differences with those patients from western countries [16, 17]. It is to be noted that we had used the recommended dosing regimen of trifluridine/tipiracil, which is 35 mg/m^2^/dose orally twice daily on days 1 through 5 and days 8 through 12 of each 28-day cycle [18]. For our knowledge, it is not known if increasing or decreasing this recommended dose could affect the patients' outcomes [18, 19]. The FTD/TPI therapy has also been reported to show higher clinical efficacy in terms of tumor response and disease control than regorafenib [20]. The FTD persists longer in tumors than in bone marrows. This can contribute a sustained antitumor effect with reduced toxicity in the patients [21]. Trifluridine is a thymidine-based nucleoside analog that is metabolized to the triphosphate metabolite, which is then incorporated into DNA, resulting in inhibition of DNA synthesis and function. The trifluridine monophosphate inhibits thymidylate synthase (TS), which is the key enzyme that provides for the sole intracellular source of thymidylate, an essential nucleotide precursor for DNA biosynthesis. Tipiracil is a thymidine phosphorylase (TP) inhibitor, which inhibits trifluridine degradative metabolism by TP; this inhibition leads to enhanced activation of trifluridine to the monophosphate and triphosphate cytotoxic metabolites [22].

Our cohort had worse overall health status; 8%, 53%, 36%, and 3% of the patients had ECOG PS of 0, 1, 2, and 3, respectively, whereas in the RECOURSE study, 56% and 44% patients had ECOG PS of 0 and 1, respectively. Although the role of ECOG PS as a prognostic factor in mCRC was considered [23], the performance had no correlation or effect on the disease outcome.

Our survival analysis revealed some interesting results. Worse PFS was significantly associated with >55 years of age and the presence of lymph node metastasis; however, a better OS was significantly associated with patients with low number of metastatic lesions and in those with higher grade of neutropenia after treatment with FTD/TPI. Neutropenia was observed in almost half of the cohort, and > 50% had neutropenia>grade 1; however, there was no report of febrile neutropenia, and this may explain the higher OS compared with that in the RECOURSE study. Although the exact mechanism underlying the association of neutropenia and improved OS is not clear, a retrospective analysis showed that neutropenia could be a good surrogate marker for adequate FTD/TPI exposure and efficacy [24]. The association between a better OS and the development of neutropenia after receiving the first cycle of FTD/TPI is in agreement with the report of Yoshino and colleagues. The authors concluded that patients who were treated with FTD/TPI and developed chemotherapy-induced neutropenia had improved OS and PFS, compared with those in the placebo group who did not develop chemotherapy-induced neutropenia [25].

Interestingly, the survival outcome in the current study was higher than that reported in the RECOURSE and TERRA trials. These differences may be attributed to several factors. First, there were discrepancies in the survival time calculations (time from the start of treatment versus time from randomization). Second, differences in the study population could exist, as only patients who received at least one dose of FTD/TPI were included in our analysis and evaluation, whereas in the RECOURSE study, at least two cycles were needed for evaluation. Moreover, the loss to follow-up of some patients in our cohort might have contributed to the survival difference. Lastly, ethnic differences and the small number of enrolled patients may have impacted the results [16, 17]. The current study supports other compassionate use programs of FTD/TPI worldwide, which have been conducted for refractory mCRC and have confirmed their efficacy in a real-world population [26–28]. Real-world data are very important and reflect the efficacy of drugs in clinical practice, but it has the limitation of being retrospective, in a less homogeneous population, and generated from uncontrolled trials with different methodologies, which could hamper the generalizability of the results [17]. These limitations have significantly challenged physicians in establishing management plans [29, 30].

In clinical settings, mCRC patients can present with secondary disease progressions. Therefore, scientists have explored the efficacy profiles of several chemotherapeutic agents in treating the refractory cases of mCRC. Bevacizumab [31], traztuzumab [32], and regorafenib [33] have shown significant potential beyond second-line setting. Data from RECOURSE and ongoing PRECONNECT trial, however, suggest FTD/TPI can be used as rechallenge therapy in refractory mCRC patients [34]. The safety and efficacy profiles of FTD/RPI are better than other available chemotherapeutic agents [17, 34]. Our findings are in line with these studies that show FTD/TPI can be used as an effective third-line treatment of mCRC.

In summary, we found that FTD/TPI had a clinical activity and fair overall response, when administered beyond the second line (given as a third line in 41% of patients and beyond the third line in 59% of patients), in a well-defined population of Saudi Arabian patients. The impact of different variables in predicting survival was validated using multivariate and regression analyses. To obtain more information about FTD/TPI in patients with mCRC, further research is needed with a larger number of patients and longer follow-up period.

The current study had some limitations, including its retrospective nature, small number of enrolled patients, and lack of data reflecting the safety profile of FTD/TPI. Despite these limitations, the current study sheds light on the real-life efficacy and outcomes of FTD/TPI among Saudi Arabian patients with mCRC. Moriwaki et al. have developed the scoring system for evaluating the survival benefit of FTD/TPI [35]. The scoring system can be adopted for further evaluation of these chemotherapeutic agents in Saudi population.

## 5. Conclusions

FTD/TPI treatment in daily clinical practice is feasible and effective. Worse PFS was significantly associated with advanced disease stage, the presence of lymph node and bone metastases, and the presence of more than two sites of metastasis. Better OS was observed in patients who had received prior adjuvant chemotherapy, patients who developed neutropenia after the first cycle of FTD/TPI, and those with an increasing number of FTD/TPI cycles. Differences in the patient characteristics between our study population and those of previous studies should be taken into consideration when interpreting survival outcomes. The use of FTD/TPI in refractory mCRC with a large number of metastatic sites may worsen prognosis. More studies to identify the predictive factors for disease outcomes are therefore necessary. Further studies in this regard can also help reduce the number of patients who would be unnecessarily exposed to toxicity.

## Figures and Tables

**Table 1:** Demographic and tumor characteristics of patients.

Characteristic	Number (%)
Age (years)	
Mean ± standard deviation	55.4 ± 11.8
Median	56.0
Sex	
Female	42 (42)
Male	58 (58)
ECOG PS	
PS 0	8 (8)
PS 1	53 (53)
PS 2	36 (36)
PS 3	3 (3)
Histopathology type	
Adenocarcinoma (NOS)	89 (89)
Mucinous adenocarcinoma	10 (10)
Signet ring adenocarcinoma	1 (1)
Site of primary cancer	
Right-side	24 (24)
Left-side	76 (76)
Stage at diagnosis	
I	2 (2)
II	5 (5)
III	29 (29)
IV	64 (64)
*KRAS* status	
Wild	44 (44)
Mutant	66 (66)
Site of metastasis	
Liver	51 (51)
Lung	41 (41)
Lymph nodes	32 (32)
Peritoneum	18 (18)
Number of metastatic sites	
1 site	21 (21)
2 sites	33 (33)
>2 sites	46 (46)
Number of metastatic lesions	
1–2 lesions	11 (11)
3–5 lesions	26 (26)
>5 lesions	63 (63)
Lines of chemotherapy and biologic agents	
Oxaliplatin	100 (100)
Irinotecan	100 (100)
Capciabine	5 (5)
5FU/leucovorin	2 (2)
Regorafenib	10 (10)
Cetuximab	25 (25)
Panitumumab	3 (3)
Bevacizumab	49 (49)

ECOG PS: Eastern Cooperative Oncology Group performance status; NOS: not otherwise specified; Ad: adenocarcinoma; right-side: ascending+transverse colon; left-side, descending colon+Sigmoid+rectum.

**Table 2:** Response to treatment with trifluridine/tipiracil (FTD/TPI); *n* = 107.

Response to FTD/TPI treatment	Number (%)
Patients not evaluated	7 (7)
Complete response (CR)	1 (1)
Partial response (PR)	3 (3)
Stable disease (SD)	28 (28)
Progressive disease (PD)	68 (68)

**Table 3:** Cox hazard regression analysis of the independent progression-free survival (PFS) predictors.

	*P* value	HR^∗^	95% CI^∗∗^	
			Lower	Upper
Age (≥55 years)	0.719	1.024	0.647	1.622
Sex (male)	0.647	0.982	0.774	1.237
Advanced disease stage (III–IV)	0.037	2.614	1.102	7.524
Prior adjuvant therapy	0.037	0.892	0.481	0.994
Lymph node metastasis	0.018	3.664	1.187	8.650
Bone metastasis	0.036	2.790	1.073	8.221
Number of metastatic sites (>2)	0.020	1.723	1.089	2.727
Duration since diagnosis (>30 months)	0.442	1.187	0.767	1.837
Neutropenia after first cycle of FTD/TPI	0.041	0.738	0.425	0.924
Baseline absolute eosinophil count	0.022	2.089	1.027	4.910
Number of FTD/TPI cycles	0.006	0.899	0.833	0.969

^∗^HR: hazard ratio; ^∗∗^CI: confidence interval; FTD/TPI: trifluridine/tipiracil.

**Table 4:** Mortality predictors among the studied cohort: logistic regression analysis.

Variable	Multivariate	
	OR (95% CI)	*P* value
Age (≥55 years)	1.675 (0.755–3.718)	0.205
Sex (male)	0.804 (0.361–1.787)	0.592
Advanced disease stage (III–IV)	2.245 (1.014–5.161)	0.048
Prior adjuvant chemotherapy	0.926 (0.408–0.991)	0.045
Lymph node metastasis	2.465 (1.078–5.638)	0.033
Liver metastasis	2.906 (1.115–7.579)	0.029
Number of metastatic sites (≥2)	1.554 (1.041–2.618)	0.045
Duration since diagnosis (≥30 months)	0.590 (0.258–0.804)	0.036
Neutropenia after first cycle of FTD/TPI	0.597 (0.269–0.954)	0.046
Number of FTD/TPI cycles	0.832 (0.707–0.978)	0.026

OR: odds ratio; CI: confidence interval; FTD/TPI: trifluridine/tipiracil.

**Table 5:** Cox hazard regression of the independent overall survival predictors.

	*P* value	HR^∗^	95% CI^∗∗^	
			Lower	Upper
Age (≥55 years)	0.046	1.667	1.097	3.100
Sex (male)	0.457	0.891	0.656	1.208
Stage (III–IV)	0.044	1.283	1.035	2.940
Prior adjuvant chemotherapy	0.037	0.892	0.481	0.994
Lymph node metastasis	0.147	1.514	0.865	2.650
Liver metastasis	0.025	2.015	1.091	3.720
Number of metastatic sites (≥2)	0.038	1.248	1.036	1.846
Duration since diagnosis (≥30 months)	0.145	0.992	0.974	1.011
Neutropenia after first cycle of FTD/TPI	0.039	0.633	1.064	2.167
Number of FTD/TPI cycles	0.002	0.769	0.664	0.891

^∗^HR: hazard ratio; ^∗∗^CI: confidence interval; FTD/TPI: trifluridine/tipiracil.

## Data Availability

All data generated or analyzed during this study are included in this published article.

## Abbreviations

CR: Complete response
ECOG: Eastern Cooperative Oncology Group
FTD/TPI: Trifluridine/tipiracil
HER2: Human epidermal growth factor receptor
mCRC: Metastatic colorectal cancer
OS: Overall survival
PD: Progressive disease
PFS: Progression-free survival
PR: Partial response
PS: Performance status
RECIST: Response evaluation criteria in solid tumors
SD: Stable disease.

## Supplementary Materials

Supplementary 1 supplementary file for Lonsurf 15 mg SFDA Registration Certificate.

Supplementary 2 supplementary file for Lonsurf 20 mg SFDA Registration Certificate.
