# Supplementary material for: Outcomes of Patients with Metastatic Colorectal Cancer Treated with Trifluridine/Tipiracil beyond the Second Line: A Multicenter Retrospective Study from Saudi Arabia
Source: J Oncol. 2022 Sep 12;2022:3796783. doi: 10.1155/2022/3796783 (PMC9485708; doi:10.1155/2022/3796783)
Supplement: Supplementary 1 — supplementary file for Lonsurf 15 mg SFDA Registration Certificate. [file 3796783.f1.pdf]

المكرم مدير الشؤون التنظيمية لشركة ستكو فارما المحترم

السلام عليكم ورحمة الله وبركاته،،،

إشارة إلى خطابكم الوارد برقم ١٥٩٠٥/ع بتاريخ ١٤٤٠/٠٣/١٠هـ والمتضمن تسديد المقابل المالي لإصدار شهادة تسجيل المستحضر التالي:

Trade name	Registration no
Lonsurf 15 mg/6.14 mg Film Coated Tablet	1-5193-18

عليه تجدون بالمرفق شهادة تسجيل المستحضر أعلاه.

مع أطيب تحياتي،،،

رئيس قسم تراخيص الأدوية البشرية

عدنان بن دخيل الصاعدي

3292 Northern Ring Road – An nafal District Riyadh 13312-6288 www.sfda.gov.sa e-mail:Drug-Dept@sfda.gov.sa
٣٢٩٢ الطريق الدائري الشمالي - حي النفل الرياض ١٣٣١٢-٦٢٨٨

شهادة تسجيل مستحضر صيدلاني
Registration Certificate of a Pharmaceutical Product

Certificate No:	2018/678	رقم الشهادة:
Issue date:	2018-11-21	تاريخ الاصدار:

Valid for 5 years of the date of issuance — صالحة لغاية خمس سنوات من تاريخ الإصدار

**Trade name**	LONSURF 15MG/6.14MG F.C.TABLET	**الاسم التجاري**
**Registration No.**	1-5193-18	**رقم التسجيل**
**Generic name & Strength/unit**	TRIFLURIDINE 15MG + TIPIRACIL 6.14MG FILM COATED TABLET	**الاسم العلمى والتركيز**
**Marketing company – Registration No.**	لس لابوراتوريز سيرفيه - الفرنسية 5193	**الشركة المسوقة ورقم تسجيلها**
**Manufacturing company – Registration No.**	تايهو فارماسيوتيكال كو ليمتد كيتاجيما بلانت - اليابانية 5194	**الشركة الصانعة ورقم تسجيلها**
**Agent**	(الشركةالسعوديةالعالميةللتجارة (ستكو	**وكيل البند**
**Dosage form**	FILM COATED TABLET	**الشكل الصيدلانى**
**Package size/Volume**	20 قرص	**حجم العبوة**
**Shelf-life**	30 شهرا	**مدة الصلاحية**
**Storage conditions**	يحفظ عند درجة حرارة أقل من 30 درجة مئوية	**ظروف التخزين**

Legal status — **طريقة الوصف**

- وصفة طبيـة عن طريق (طبيب عام [ ] استشارى [ ] اخصائى [X]) - وصفة ادوية مخدرة [ ] - وصفة أدوية نفسية [ ] - بدون وصفة طبيـة [ ]

By medical prescription (G.P [ ] Consultant [ ] specialist [X]) By Narcotic prescription [ ] By Psychiatric prescription [ ] OTC [ ]

For Hospital use only [X] Controlled [ ] — خاضع للرقابه [ ] للمستشفيات فقط [X]

**CIF/Ex-factory price (S.R)**	2143.125	**سعر التصدير/ المصنع ر.س**
**Public price (S.R)**	2593.20	**سعر الجمهور ر.س**
**Price category**	3	**رقم شريحة السعر**
**Note**		**ملاحظـــــات**

Based on the approval of the Executive President of the Saudi Food & Drug Authority on the decision taken by the Registration Committee of Medicinal Products & Manufacturers at its meeting
No: 956 **Dated:** 23-12-1439
The above product is registered & priced at SFDA
Note
Manufacturing company shall print on each package the following information:
1-Public Price 2- Registration No
3- Batch No 4-Production and Expiry Date
5- Trade Name (in Arabic).
6- Storage Conditions(in Arabic).

بناءا على اعتماد معالي الرئيس التنفيذي للهيئة العامة للغذاء والدواء لقرار لجنة تسجيل شركات الأدوية ومنتجاتها في جلستها
رقم : 956 و تاريخ : 1439-12-23
تم تسجيل و تسعير المنتج بالمواصفات الموضحة أعلاه
ملاحظة
تقوم الشركة الصانعة بطباعة ما يلي على كل عبوة :
1- سعر الجهور 2- رقم التسجيل
3- رقم التشغيلة 4- تاريخ الصنع والإنتهاء
5- الاسم التجاري باللغة العربية
6- ظروف التخزين باللغة العربية

رئيس قسم تراخيص الأدوية البشرية
Head of Human Drug Licensing

عدنان بن دخيل الصاعدي
Adnan D. Alsaedi

Printed By: SFDA\HGHarbi

83369
