# Supplementary material for: Outcomes of Patients with Metastatic Colorectal Cancer Treated with Trifluridine/Tipiracil beyond the Second Line: A Multicenter Retrospective Study from Saudi Arabia
Source: J Oncol. 2022 Sep 12;2022:3796783. doi: 10.1155/2022/3796783 (PMC9485708; doi:10.1155/2022/3796783)
Supplement: Supplementary 2 — supplementary file for Lonsurf 20 mg SFDA Registration Certificate. [file 3796783.f2.pdf]

الهيئة العامة للغذاء والدواء
Saudi Food & Drug Authority
رقم الصـادر : 21075/ع
تاريخ الصـادر : 14 / 03 / 1440 هـ
نوع المرفق : لا يوجد
الإحالة : الشركة السعودية العالمية للتجا
2-1440-1-21075-96-2

بسم الله الرحمن الرحيم

المملكة العربية السعودية
الهيئة العامة للغذاء والدواء
( ٢٥٥ )
قطاع الدواء

**المكرم مدير الشؤون التنظيمية لشركة ستكو فارما          المحترم**

**السلام عليكم ورحمة الله وبركاته،،،**

إشارة إلى خطابكم الوارد برقم ١٥٩٠٤/ع بتاريخ ١٠/٠٣/١٤٤٠هـ والمتضمن تسديد المقابل المالي لإصدار شهادة تسجيل المستحضر التالي:

| Trade name | Registration no |
|---|---|
| Lonsurf 20 mg/8.19 mg Film Coated Tablet | 2-5193-18 |

عليه تجدون بالمرفق شهادة تسجيل المستحضر أعلاه.

**مع أطيب تحياتي،،،**

**رئيس قسم تراخيص الأدوية البشرية**

**عدنان بن دخيل الصاعدي**

الهيئة العامة للغذاء والدواء قطاع الدواء

**Drug Sector Saudi Food & Drug Authority**

# شهادة تسجيل مستحضر صيدلاني

# Registration Certificate of a Pharmaceutical Product

| | | |
|---|---|---|
| Certificate No: | 2018/679 | رقم الشهادة: |
| Issue date: | 2018-11-21 | تاريخ الاصدار: |

Valid for 5 years of the date of issuance صالحة لغاية خمس سنوات من تاريخ الإصدار

| | | |
|---|---|---|
| **Trade name** | LONSURF 20MG/8.19MG F.C.TABLET | **الاسم التجاري** |
| **Registration No.** | 2-5193-18 | **رقم التسجيل** |
| **Generic name & Strength/unit** | TRIFLURIDINE 20MG + TIPIRACIL 8.19MG FILM COATED TABLET | **الاسم العلمى والتركيز** |
| **Marketing company – Registration No.** | لس لابوراتوريز سيرفيه - الفرنسية 5193 | **الشركة المسوقة ورقم تسجيلها** |
| **Manufacturing company – Registration No.** | تايهو فارماسيوتيكال كو ليمتد كيتاجيما بلانت - اليابانية 5194 | **الشركة الصانعة ورقم تسجيلها** |
| **Agent** | (الشركةالسعوديةالعالميةللتجارة (ستكو | **وكيل البند** |
| **Dosage form** | FILM COATED TABLET | **الشكل الصيدلانى** |
| **Package size/Volume** | 20 قرص | **حجم العبوة** |
| **Shelf-life** | 30 شهرا | **مدة الصلاحية** |
| **Storage conditions** | يحفظ عند درجة حرارة أقل من 30 درجة مئوية | **ظروف التخزين** |

**Legal status** **طريقة الوصف**

- وصفة طبيـة عن طريق (طبيب عام ☐ استشارى ☐ اخصائى ☒ ) - وصفة ادوية مخدرة ☐ - وصفة أدوية نفسية ☐ - بدون وصفة طبيـة ☐

By medical prescription ( G.P ☐ Consultant ☐ specialist ☒ By Narcotic prescription ☐ By Psychiatric prescription ☐ OTC ☐

For Hospital use only ☒ Controlled ☐ خاضع للرقابه ☐ للمستشفيات فقط ☒

| | | |
|---|---|---|
| **CIF/Ex-factory price (S.R)** | 2767.087 | **سعر التصدير/ المصنع ر.س** |
| **Public price (S.R)** | 3348.15 | **سعر الجمهور ر.س** |
| **Price category** | 3 | **رقم شريحة السعر** |
| **Note** | | **ملاحظـــات** |

Based on the approval of the Executive President of the Saudi Food & Drug Authority on the decision taken by the Registration Committee of Medicinal Products & Manufacturers at its meeting

**No:** 956 **Dated:** 23-12-1439

The above product is registered & priced at SFDA

**Note**

Manufacturing company shall print on each package the following information:

1-Public Price 2- Registration No

3- Batch No 4-Production and Expiry Date

5- Trade Name (in Arabic).

6- Storage Conditions( in Arabic).

بناءا على اعتماد معالي الرئيس التنفيذي للهيئة العامة للغذاء والدواء لقرار لجنة تسجيل شركات الأدوية ومنتجاتها في جلستها

رقم : 956 و تاريخ : 1439-12-23

تم تسجيل و تسعير المنتج بالمواصفات الموضحة أعلاه

**ملاحظة**

تقوم الشركة الصانعة بطباعة ما يلي على كل عبوة :

1- سعر الجهور 2- رقم التسجيل

3- رقم التشغيلة 4- تاريخ الصنع والإنتهاء

5- الاسم التجاري باللغة العربية

6- ظروف التخزين باللغة العربية

رئيس قسم تراخيص الأدوية البشرية

**Head of Human Drug Licensing**

عدنان بن دخيل الصاعدي

**Adnan D. Alsaedi**

Printed By: SFDA\HGHarbi

3292 Northern Ring Road – An nafal District Riyadh 13312-6288 www.sfda.gov.sa e-mail:Drug-Dept@sfda.gov.sa

٣٢٩٢ الطريق الدائري الشمالي - حي النفل الرياض ١٣٣١٢-٦٢٨٨



Any scrape or alteration to the ceritificate is considered invalid أي كشط أو تعديل على الشهادة تعتبر لاغية
